# Photosensitivity From Avapritinib: Pharamacovigilance Analysis

**DOI:** 10.2196/39229

**Published:** 2022-08-10

**Authors:** Ajit Venkatakrishnan, Brandon Chu, Pushkar Aggarwal

**Affiliations:** 1 University of Cincinnati Cincinnati, OH United States; 2 University of Maryland College Park, MD United States; 3 College of Medicine University of Cincinnati Cincinnati, OH United States

**Keywords:** oncology, Avapritinib, drug-induced, adverse reaction, photosensitizer, photosensitizing, cancer, pharmacovigilance, pharmaceutical, photosensitive, photosensitivity, light, adverse event, side effect, tumor, pharmacology

## Abstract

Certain protein kinase inhibitors have been reported to cause photosensitivity. Avapritinib is a tyrosine kinase inhibitor that was approved in January 2020. The aim of this analysis was to determine if a statistically significant signal exists between Avapritinib and photosensitivity in the real-world population. A disproportionality analysis was conducted using the Food and Drug Administration Adverse Event Reporting System (FAERS) from January 1, 2020, to December 31, 2021. A literature review was also performed to identify case reports of Avapritinib-induced photosensitivity. A total of 13 adverse event reports with Avapritinib as the drug and photosensitivity as the reaction were identified in FAERS. Avapritinib was the suspect drug in all 13 reports, and in 12 of the 13 reports, Avapritinib was the only drug listed. Disproportionality analysis found a proportional reporting ratio of 11.0, *χ*^2^_1_=107, reporting odds ratio of 11.0, and a lower limit of the 95% CI of the information component of 2.1. The literature review found 1 case report of Avapritinib-induced photosensitivity in a patient who had been taking Avapritinib 300 mg daily for 5 months. A statistically significant signal was found between Avapritinib use and photosensitivity. Clinicians should continue to balance the benefits and risks when prescribing Avapritinib to patients.

## Introduction

Humans have been exposed to UV light for millions of years. This exposure has beneficial effects in increasing vitamin D levels and in treating psoriasis, vitiligo, atopic dermatitis, and scleroderma among others [1]. However, this same UV light can increase the risk of skin carcinoma, cataracts, and age-related macular degeneration. Certain drugs have been found to increase the sensitivity of the skin to sunlight. These drugs are categorized as sun-sensitizing drugs and can lead to drug-induced photosensitivity. Drug-induced photosensitivity can present as erythema and can progress to blisters, bullae, and severe pain. Knowing which drugs can lead to drug-induced photosensitivity is paramount so that clinicians can adequately advise patients on sun protection and reduce the risk of skin cancer.

Certain protein kinase inhibitors such as Vemurafenib, Vandetanib, and Imatinib have been reported to cause photosensitivity [2,3]. Avapritinib is a tyrosine kinase inhibitor that was approved in January 2020 and is used for the treatment of systemic mastocytosis and unresectable or metastatic gastrointestinal stromal tumor. Because Avapritinib has been in the market for such a short period of time, adverse reactions attributed to the drug are still being discovered. In 2021, the Food and Drug Administration (FDA) issued an alert that they are evaluating the need for regulatory action on the potential signal of photosensitivity from Avapritinib [4]. The objective of this analysis was to determine if a statistically significant signal exists between Avapritinib and photosensitivity in the real-world population.

## Methods

Adverse event reports from the FDA Adverse Event Reporting System (FAERS) [5] from January 1, 2020, to December 31, 2021, were downloaded. Reports were filtered to those with the drug Avapritinib and the MedDRA [6] term photosensitivity reaction. Reports were further filtered to those with Avapritinib as the suspect drug, and duplicate cases were removed. Disproportionality analysis was performed to identify if a significant signal exists between the drug and adverse event of interest. Statistical analysis was carried out in SAS [7] version 9 (SAS Institute). A literature review using PubMed [8] was performed to identify case reports of Avapritinib-induced photosensitivity.

## Results

A total of 13 adverse event reports with Avapritinib as the drug and photosensitivity as the reaction were identified in FAERS with the earliest report in May 2020 and the latest in November 2021. The most common coreported events were edema, increased lacrimation, fatigue, rash, abdominal discomfort, and diarrhea. Avapritinib was the suspect drug in all 13 reports, and in 12 of the 13 patients, Avapritinib was the only drug listed. In the other case report, the patient was taking insulin glargine, insulin aspart, ondansetron, diphenhydramine, loratadine, loperamide, bisacodyl, and tramadol in addition to Avapritinib. All 13 reports originated from the United States. In addition, in 5 cases, the adverse event resulted in death, a life-threatening condition, hospitalization, disability, congenital anomaly, or other serious condition. However, the case reports do not specify the cause of the above serious conditions. It may be related to photosensitivity, the underlying condition for which the patient was being treated, or another unknown cause. The average age of the patients was 60 years with a range of 31 to 80 years. A total of 11 patients were men, and the remaining 2 were women. The indication for the use of Avapritinib was gastrointestinal stromal tumor in 9 of the patients and systemic mastocytosis in the remaining 5 (Table 1). Disproportionality analysis found a proportional reporting ratio (PRR) of 11.0, *χ*^2^=107, reporting odds ratio (ROR) of 11.0, and the lower limit of a 95% CI of the information component (IC_025_) of 2.1.

The signal between Avapritinib and photosensitivity was statistically significant based on each of the following three criteria:

1. PRR≥2, chi-square ≥4, and number of events ≥3 [9]
2. ROR>1 [10]
3. IC_025_>0 [11]

**Table 1:** Demographic data of patients with Avapritinib use and photosensitivity reaction.

		Cases of Avapritinib and photosensitivity reaction (N=13), n (%)
**Gender**		
	Male	11 (85)
	Female	2 (15)
**Age (years)**		
	31-50	3 (23)
	51-60	2 (15)
	61-70	5 (38)
	71-80	2 (15)
	Unknown	1 (8)
**Indication for use of Avapritinib**		
	Gastrointestinal stromal tumor	9 (69)
	Systemic mastocytosis	4 (31)
**Seriousness**		
	Resulted in death, a life-threatening condition, hospitalization, disability, congenital anomaly, or other serious condition	5 (38)
	Did not result in above	8 (62)

## Discussion

The literature review found 1 case report of Avapritinib-induced photosensitivity [12]. This patient was a 56-year-old female who was being treated for a stage IV gastrointestinal stromal tumor with Avapritinib. She presented with a rash that initially appeared as a sunburn and progressed to the development of bullae and pain. Histopathology identified dermal edema, mixed inflammatory infiltrates, rare dyskeratotic keratinocytes, and follicular interface. The patient had been on Avapritinib 300 mg daily for 5 months when the rash first occurred. The patient was diagnosed with Avapritinib-induced photosensitivity. Avapritinib was permanently discontinued, and 0.1% triamcinolone cream was initiated with improvement in the rash. Further, nonclinical findings of phototoxicity with Avapritinib use were found in vitro mouse fibroblasts and in vivo rat studies [13]. The European Medicines Agency lists a warning of photosensitivity with Avapritinib and a 1.1% incidence of photosensitivity during clinical trials [14].

The pathophysiology behind the photosensitivity from Avapritinib has not been fully elucidated but may share a similar mechanism to the cutaneous toxicities of other tyrosine kinase inhibitors such as imatinib. For example, Imatinib inhibits activity of the c-KIT gene leading to hypopigmentation and reduced protection against UV exposure [2]. Similarly, Avapritinib is also a potent inhibitor of the KIT gene [15]. Further studies are needed to identify the pathophysiology underlying this possible reaction.

FAERS provides a passive pharmacovigilance risk signal and does not by itself demonstrate causal associations. The adverse event may be a result of the drug, the underlying disease, or a combination of the two. Individual case causality assessments, periodic aggregate assessment of available clinical safety data, and well-designed randomized controlled clinical trials are needed to validate the safety signal and to assess for an association between an adverse event and a drug [16]. In addition, not every adverse event is reported to the FDA and thus incidence of the adverse event cannot be calculated. Further, the time to onset of the adverse event from initiation of the drug is not provided in FAERS. If there is a long latency period to the development of the adverse event, the benefit of the drug may be more likely to supersede the risk. However, FAERS has advantages in identifying signals in a large and diverse patient group in the real world that are not always identified in the early clinical trials [17,18].

A statistically significant signal was found between Avapritinib use and photosensitivity. Of these adverse event reports of Avapritinib and photosensitivity, 85% (n=11) of the reports were in male patients and 15% (n=2) in female patients. Further studies are needed to evaluate whether the disproportionality signal between Avapritinib and photosensitivity represents a causal association. Clinicians should continue to balance the benefits and risks when prescribing Avapritinib to patients.

## Abbreviations

FAERS: Food and Drug Administration Adverse Event Reporting System
FDA: Food and Drug Administration
IC_025_: lower limit of a 95% CI of the information component
PRR: proportional reporting ratio
ROR: reporting odds ratio
